# Dimethyl sulfoxide–induced DNA demethylation during vitrification of early cleavage-stage embryos and possible countermeasures

**DOI:** 10.1007/s10815-025-03415-7

**Published:** 2025-02-05

**Authors:** M. Shida, J. Ito, Y. Inoue, S. Hara, K. Shirasuna, H. Iwata

**Affiliations:** Deportment of Animal Science, Tokyo University of Agriculture, Funako 1737, Atsugi City, Japan

**Keywords:** N-acetyl-l-cysteine, Dimethyl sulfoxide, DNA methylation, Propylene glycol, Vitrification

## Abstract

**Purpose:**

Dimethyl sulfoxide (DMSO) alters DNA methylation in vitrified-warmed embryos and potentially affects subsequent development. This study aimed to examine possible countermeasures against DMSO-induced demethylation.

**Methods:**

In vitro-produced bovine embryos (8-cell stage) were vitrified using a combination of DMSO and ethylene glycol (EG) or propylene glycol (PG) + EG. After warming, the lipid content and expression levels of 5-methylcytosine (5mC), 5-hydroxymethylcytosine (5hmC), DNMTs, and TETs were examined. In addition, RNA-sequencing was performed on blastocysts derived from the vitrified embryos. Furthermore, the effect of supplementation with a vitrification medium containing DMSO and N-acetyl-l-cysteine (NAC, 5 mM) on the levels of 5mC in embryos was examined.

**Results:**

Vitrification decreased the levels of 5mC and increased the levels of 5hmC in 8-cell stage embryos. Low levels of 5mC persisted until the blastocyst stage in the DMSO group but increased in the PG group. The expression level of TET3A was higher in the DMSO group than in the fresh group, but not in the PG group. Both cryoprotectants reduced the lipid levels in post-warmed 8-cell stage embryos. The addition of NAC ameliorated DMSO-induced demethylation at both the 8-cell and blastocyst stages. RNA-seq analysis revealed that PG-specific pathways included ribosomes and mitochondria and that both DMSO and PG affected cGMP-PGK, MAPK, Wnt, and insulin secretion–related signaling. The *K-medoids* method predicted that DMSO affected cell adhesion molecules and that MAPK signaling was affected the most.

**Conclusions:**

PG and NAC may antagonize DMSO-induced demethylation; however, PG exerts adverse effects on embryos.

## Introduction

Vitrification is a widely used assisted reproductive technology that increases chances of conception in humans. Vitrification requires high concentrations of cellular membrane-impermeable and permeable cryoprotectants, and dimethyl sulfoxide (DMSO) has been used as a major cryoprotectant. Although vitrification is an embryo- and oocyte-friendly technique, it induces osmotic shock and oxidative stress, which affect the cellular cytoskeleton, organelles, and DNA integrity [1–4], resulting in reduced developmental ability of embryos with upregulated expression of DNA repair gene [5]. Accumulating evidences has shown that the birth weight of offspring derived from vitrified-warmed embryos is greater than that of fresh embryos in humans and rats [6–8], suggesting that vitrification induces epigenetic changes in post-warmed embryos [9]. Consistently, 5-methylcytosine (5mC) levels in the 8-cell and blastocysts stages of vitrified-warmed mouse embryos and the expression levels of imprinted genes were lower than those from fresh embryos [10].

Weihong et al. reported that vitrification with DMSO decreased the levels of 5mC in bovine oocytes; however, the use of propylene glycol (PG) instead of DMSO increased these levels [11]. In this context, Pollock et al. [12] reported that replacing DMSO with an osmolyte-based solution diminished the increase in the levels of 5-hydroxymethylcytosine (5hmC) in mesenchymal stromal cells following vitrification. Moreover, DMSO affects the expression of enzymes such as methyltransferases (DNMTs) and demethylases (TETs) in embryos [13]. DMSO, an oxidant and a source of oxygen, is potentially involved in the oxidation of methylene residues in DNA. Therefore, we hypothesized that DMSO is a causal factor for vitrification-induced demethylation and that the use of other cryoprotectants or addition of competitive molecules to the vitrification solution may hamper DMSO-induced demethylation.

Here, we aimed to vitrify bovine 8-cell stage embryos using a combination of DMSO + ethylene glycol (EG) or PG + EG and examine DNA methylation levels, DNMT and TET expression, and lipid content in vitrified-warmed embryos, along with RNA-sequence (RNA-seq) of vitrified-warmed embryos, to determine cryoprotectant-specific changes in the genetic background. Furthermore, we examined the effect of vitrification medium supplemented with DMSO and N-acetyl-l-cysteine (NAC) on vitrification-induced demethylation of 8-cell and blastocyst stage embryos.

## Materials and methods

### Oocyte collection, maturity, and fertilization

Bovine ovaries were collected from a slaughterhouse and transported to the laboratory in phosphate-buffered saline (PBS) within 3 h. Cumulus-oocyte complexes (COCs) were aspirated using an 18-gauge needle connected to a 10-mL syringe. The COCs were cultured in a 100-μL droplet of in vitro maturation medium (10 oocytes/drop) covered by paraffin oil (tissue culture grade; Nacalai Tesque) for 21 h. TCM-199 medium (Gibco, Grand Island, NY, USA) supplemented with 10% fetal calf serum (FCS) (5703H; ICN Pharmaceuticals, Costa Mesa, CA, USA), 5 mM taurine, and 10-ng/mL epidermal growth factor was used as the maturation medium. Then, the COCs were incubated with freeze-thawed semen from Japanese black cows, as described previously [14]. In vitro fertilization and in vitro culture (IVC) media were based on synthetic oviductal fluid, modified according to previous reports [15]. After fertilization, the COCs were incubated in IVC medium for 48 h in an atmosphere of 5% CO₂. Embryos cleaved over 8-cells were selected and used for experiments.

### Vitrification and warming of 8-cell embryo

Vitrification medium TCM199 (pH 7.3) containing 6.25 mM HEPES and 20% FCS was referred to as the rinsing solution (RS). Equilibration was performed in RS containing 7.5% DMSO + 7.5% EG or 7.5% PG + 7.5 EG (equilibration solution; ES) for 5 min at 38.5 °C. Vitrification was then performed in RS solution containing 15% DMSO + 15% EG or 15% PG + 15% EG (vitrification solution; VS). Embryos were treated with VS for 30 s and placed on a Cryotop device (Kitazato Crop, Shizuoka, Japan) and plunged into liquid nitrogen. for warming, the Cryotop devices with embryos was immersed directly into RS containing 1.0 M sucrose and incubated for 1 min at 38.5 °C, followed by a step wise transfer into RS containing 0.5, 0.25, and 0 M sucrose for 3, 5, and 5 min, respectively.

### Immunostaining

Vitrified-warmed embryos were incubated for 1 or 5 days and cleaved embryos or blastocyst-stage embryos that appeared morphologically viable were selected and used for immunostaining. Embryos were fixed using 4% paraformaldehyde for 1 day and permeabilized using PBS containing 0.2% Triton X-100 for 30 min, followed by blocking in PBS containing 5% bovine serum albumin for 1 h. The embryos were incubated overnight with the primary antibody, followed by treatment with secondary antibody for 1 h. For immunostaining of 5mC and 5hmC, the embryos were treated with 1 N HCl for 1 h before blocking. The embryos were then mounted on glass slides and observed under a fluorescence microscope (SCR-038447; Dianova Hamburg, Germany). To determine the expression levels of 5mC and 5hmC in the 8-cell stage, all blastomere nuclei were measured. For blastocysts, non-overlapped 5 trophectoderm (TE) cells and five inner cell mass (ICM) cells were arbitrarily selected to measure 5mC expression levels. To evaluate the expression levels of other proteins (DNMT1, DNMT3A, TET1, and TET3) in cleaved embryos, the equatorial region of the whole embryo was evaluated. The primary and secondary antibodies used are described in Table 1. In each trial, average value of control (fresh, non-vitrified embryos) is defined as 1.0, and ratio of experimental groups were calculated.

**Table 1:** Antibodies used for immunostaining

Antibody	Host	Dilution	Manufacture	Catalog No
Primary antibodies				
5mC	Rabbit	1:200	Cell Signaling, Danvers, MA, USA	D3S2Z
TET3	Rabbit	1:400	GENE Tex, Irvine, CA, USA	GTX121453
DNMT3A	Rabbit	1:200	ABCEPTA, San Diego, CA, USA	AI12955
5hmC	Mouse	1:200	GENE Tex	GTX629701
DNMT1	Mouse	1:200	Novus Biologicals, CO, USA	NB-100-56519SS
TET1	Mouse	1:200	Santa Cruz Biotechnology, TX, USA	sc-293186
Secondary antibodies				
Anti-rabit lgG (H + L)	Goat	1:500	Cell Signaling	#4412
Anti-mouse lgG(H + L)	Goat	1:500	Cell Signaling	#4408

### Assessment of lipid content in embryos

Vitrified-warmed embryos were cultured for 1 or 5 days and cleaved embryos or blastocyst-stage embryos that appeared morphologically viable were stained with Nile red (Wako, Tokyo, Japan) for 10 min. The embryos were mounted on a glass slide and the equatorial region of the embryos was imaged under a fluorescent microscope (SCR-038447; Dianova; Hamburg, Germany). The images were digitized using the ImageJ software (NIH).

### RNA-seq and analysis of the data

**Fig. 1:**
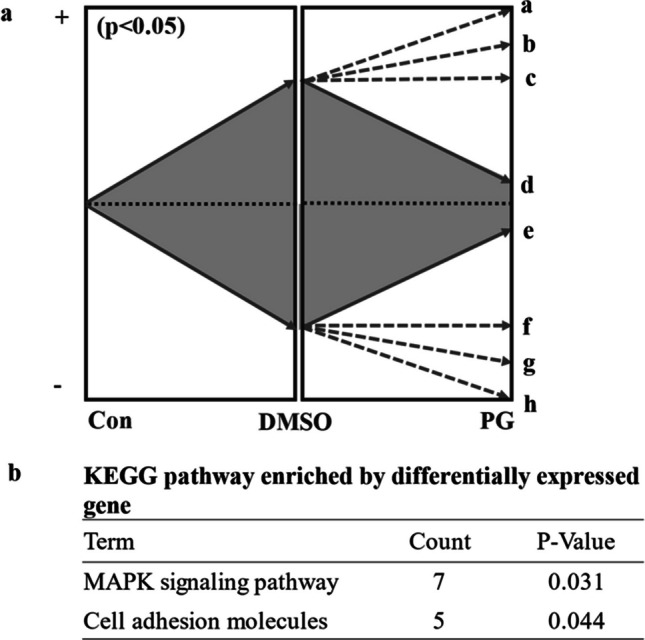
Schematic design of *K-medoids* analysis of DEGs. **a** DEGs between control and DMSO groups (*P* < 0.05) were clustered into eight groups (a–h). **a** Gene groups (d and e) having the greatest adverse directional change between DMSO and PG compared with those between control and DMSO were selected. **b** Pathway enriched by the genes groups (d, e)

In vitro-developed blastocyst-stage embryos were used for RNA-seq analysis. Embryos were vitrified, warmed at 8-cell stage, and cultured up to the blastocyst stages for 5 days. Approximately 30 blastocysts developed from fresh DMSO + EG or PG + EG 8-cell stage vitrified embryos were used for RNA extraction. RNA quality and concentration were examined using the Agilent 2100 Bioanalyzer (Agilent Technologies, Palo Alto, CA, USA). The average RNA integrity number was 9.4 ± 0.4. cDNA was produced using the NEBNext single cell/low input RNA library prep kit (New England Biolabs). The quality and quantity were determined using an Agilent 2100 Bioanalyzer, followed by re-measurement using at Kapa library quantification kit (Kapa Biosystems, Wilmington, MA, USA). Sequencing was conducted using NextSeq1000 (Illumina, San Diego, CA, USA) with single read × 100 bp. Image analysis, base calling, and quality filtering were performed using RTA version 2.4.11 (Illumina) following the manufacture’s protocol, and the sequence data were converted to Fastq using bcl2fastq2 v2.20.0.422. To prepare the sequenced data, adapter sequences, ambiguous nucleotides, and low-quality sequences were removed. The remaining sequence data were aligned to the *Bos taurus* genome sequence (ARS-UCD1.2/bosTau9) to count sequence reads. Gene expression values were evaluated as transcripts per kilobase million. The process of sequence preparation, mapping to the reference genome, and differential gene expression analysis (*P* < 0.05) were performed using the CLC Genomics Workbench ver. 22.0.2 (Qiagen, Hilden, Germany). Pathways and Gene Ontology (GO) analyses of the differentially expressed genes (DEGs) were conducted using a functional annotation tool (DAVID, https://david.ncifcrf.gov/), where the *Bos taurus* genome was used as the reference. DEGs were compared using the principal component analysis (PCA) and *K-medoids* method. The raw RNA-seq data of the blastocysts were registered in DDBJ under accession numbers PRJDB18767 and Experiment DRX576597-576,605. DEGs between the control and DMSO groups (*P* < 0.05) were clustered into eight groups based on the differences between the DMSO and PG groups using the *K-medoids* method (CLC Genomics Workbench). *K-medoids* can assign the DEGs to clusters using an algorithm [16]. In *K-medoids* clustering, DEGs are clustered into *K* separate clusters. The procedures seek to assign DEGs to cluster such that distances between DEGs of the same cluster are small while distances between clusters are large. After clustering, DEGs with the greatest differential directional changes in DMSO/control vs. PG/DMSO groups were selected (Fig. 1a) and used for the prediction of Kyoto Encyclopedia of Genes and Genomes (KEGG) pathways.

### Experimental design

In the first experiment, 8-cell stage embryos were vitrified using DMSO + EG or PG + EG. The warmed embryos were cultured to the blastocyst stage, and the rate of development was examined (29 trials with 10–12 embryos each). A total of 324, 324, and 319 8-cell stage embryos were used for control, DMSO + EG, and PG + EG groups, respectively. In addition, fresh (control) or vitrified-warmed 8-cell stage embryos were incubated for 24 h when morphologically viable embryos were subjected to immunostaining for 5mC and 5hmC. Blastocysts derived from fresh or vitrified 8-cell stage embryos were immunostained for 5mC.

In the second experiment, to determine whether vitrification using DMSO or PG affected the expression levels of DNMT1/3A and TET1/3, fresh or vitrified-warmed 8-cell stage embryos were cultured for 1 day and then subjected to immunostaining. Furthermore, warmed 8-cell stage embryos were cultured to obtain blastocysts, in which lipid content was measured.

In the third experiment, 8-cell stage embryos were vitrified using DMSO or PG, and the warmed embryos were incubated for 5 days. A comprehensive gene expression analysis was conducted using blastocysts. Thirty embryos were prepared for each group, and this was repeated three times with a differential ovary series.

In these four experiments, we hypothesized that the oxidative properties of DMSO were causal factors for demethylation. 5 mM NAC (M8H6852; Nacalai Tesque, Inc; Kyoto, Japan) was added to the equilibration and vitrification solution containing DMSO + EG. Vitrified-warmed embryos were stained for 1 and 5 days, after which the embryos were stained for 5mC.The rate of developmental into the blastocyst stage was examined (29 trials with ten embryos).

### Statistical analysis

All data were analyzed using the Shapiro–Wilk test. Three parametric datasets were analyzed using one-way analysis of variance (ANOVA), followed by Tukey’s post hoc test. Non-parametric data were analyzed using the Kruskal–Wallis and Steel–Dwass tests for multiple comparisons. Parametric data were analyzed using Student’s *t* test and non-parametric data were analyzed using the Mann–Whitney *U* test. Date with *P* < 0.05 was considered statistically significant.

## Results

### Summary of experiments including embryo staining

The present study contains many comparisons of embryos stained against proteins and lipids. Therefore, a summary of the result is shown in Table 2.

**Table 2:** Summary of experiments

Fig. No	Targets	Stage	Con	DMSO	PG
Figure 2	5mC	8-cell	1.00 a	0.75 b	0.834 c
Figure 2	5hmC	8-cell	1.00 a	1.14 b	1.16 b
Figure 3	5mC	Blastocyst	1.00 a	0.64 b	1.53 c
Figure 4a	DNMT1	8-cell	1.00 a	1.08 b	
Figure 4b	DNMT1	8-cell	1.00		0.99
Figure 4c	DNMT3A	8-cell	1.00 a	0.86 b	
Figure 4d	DNMT3A	8-cell	1.00		0.92
Figure 5a	TET1	8-cell	1.00	1.10	
Figure 5b	TET1	8-cell	1.00		1.04
Figure 5c	TET3	8-cell	1.00 a	1.08 b	
Figure 5d	TET3	8-cell	1.00		0.98
Figure 6a	Lipid	8-cell	1.00 a	1.35 b	1.23 b
Figure 6b	Lipid	Blastocysts	1.00	1.10	1.08
			NAC** − **	NAC +	
Figure 7a	5mC	8-cell	1.00 a	1.28 b	
Figure 7b	5mC	Blastocysts	1.00 a	1.17 b	

*Con* control, *DMSO* embryo vitrified with EG + DMSO, *PG* embryo vitrified with EG + PG, *NAC*** − **embryo vitrified with EG + DMSO, *NAC* + embryo vitrified with EG + DMSO + NAC 5 mM. *8-cell* e-cell stage embryos. Average of control was defined as 1.0. a-c, *P* < 0.05

### Effect of vitrification on development and 5mC levels of embryos

**Fig. 2:**
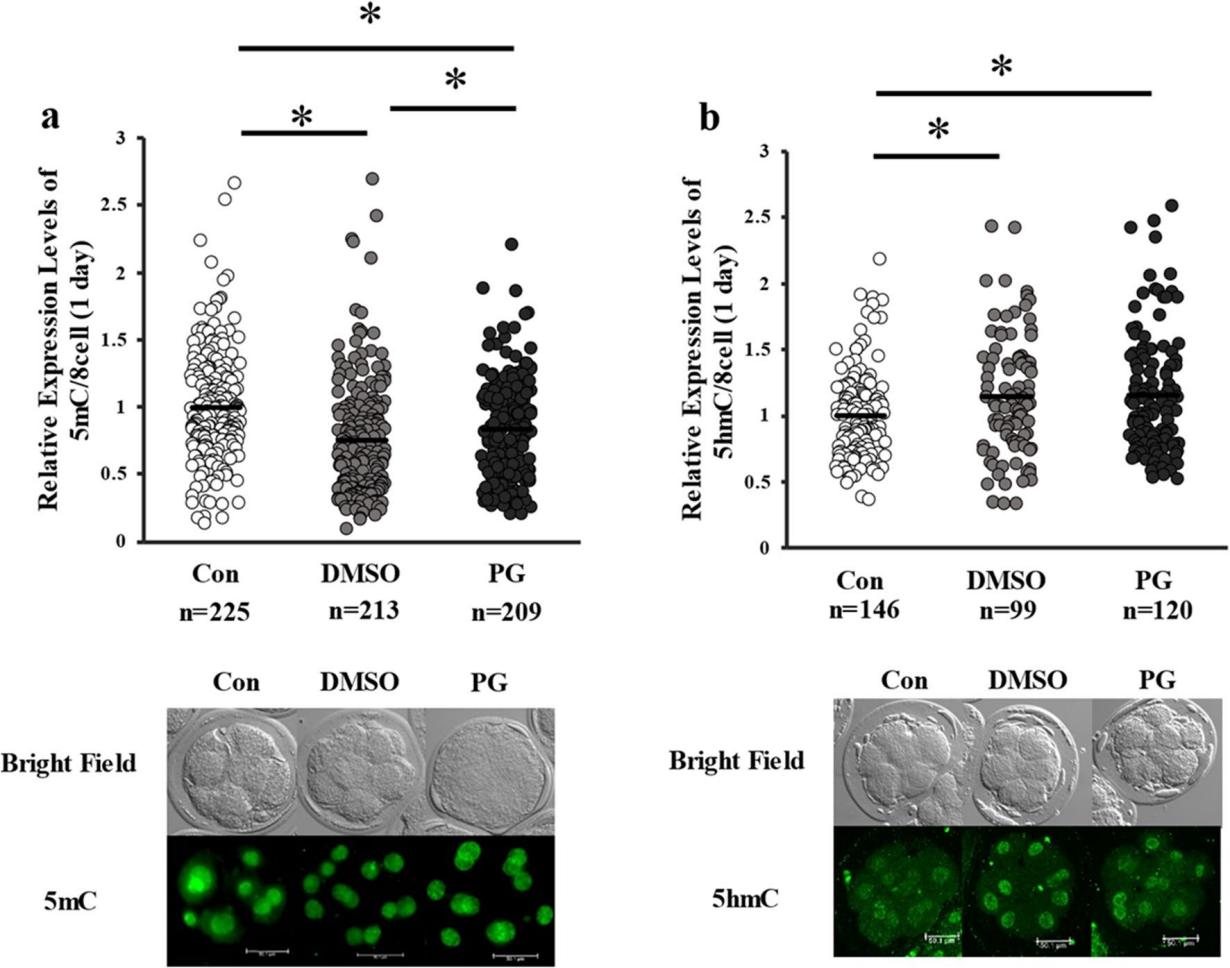
Expression levels of 5-methylcytosine (**a**) and 5-hydroxymethylcytosine (**b**) in each blastomere of fresh or vitrified-warmed embryos with DMSO + EG or PG + EG. Average of control was defined as 1.0. *, different scripts indicate significantly differences (*P* < 0.05). n indicates the number of blastomeres examined. Representative images are shown below the figures, and the bar indicates 50.1 µm. Number of embryos: 24, 23, and 25 for 5mC; 20, 13, and 17 for 5hmC. a, b: Kruskal–Wallis test was used for the comparison

**Fig. 3:**
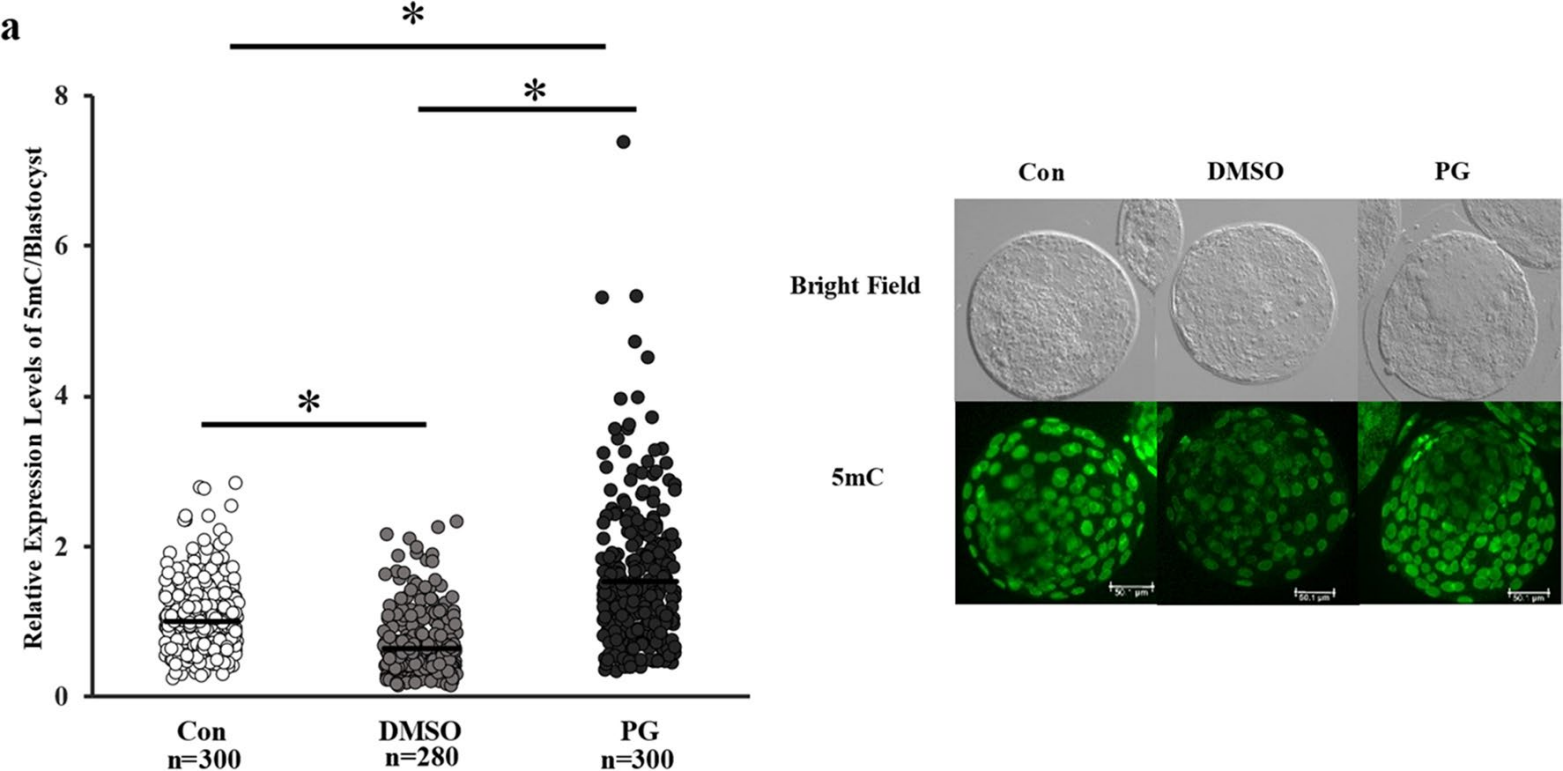
Levels of 5mC in the blastocyst-stage embryos derived from vitrified 8-cell stage embryos treated with DMSO + EG or PG + EG. Representative image is shown below the figure and the bar indicates 50.1 µm. Control indicates fresh embryos. Average of control was defined as 1.0. *n* indicates the number of blastomeres examined. *P < 0.05. Kruskal–Wallis test was used for the comparison. Number of embryos: 30, 28, and 30 for blastocysts

The rate of development of vitrified-warmed embryos to the blastocyst stage was lower than that of their fresh counterparts (*P* < 0.05); however, the total blastocyst cell numbers were comparable (Table 3). The levels of total DNA methylation at the 8-cell stage were lower in vitrified-warmed embryos (Fig. 2a), and significantly lower in the DMSO + EG group than that in the PG + EG group (Fig. 2a). Furthermore, the levels of 5hmC in the blastomeres were higher in the vitrified-warmed embryos than that in the fresh control (Fig. 2b). After subsequent incubation for 5 days, the levels of 5mC in the blastomeres of the blastocysts were significantly lower in the DMSO + EG group but higher in the PG + EG group than that in their fresh counterparts (Fig. 3).

**Table 3:** Effect of different cryoprotectants on the developmental ability of embryos

Groups	No. of	No. of	Blastocysts		
	Trials	embryos	Rate (%)	No	TCN
Control	29	324	38.0 ± 2.8	30	93 ± 6.1
DMSO	29	324	25.2 ± 3.0	28	91 ± 6.8
PG	29	319	21.4 ± 3.1	30	89 ± 5.8

Eight-stage embryos were vitrified using DMSO + EG (DMSO group) or PG + EG (PG group) Date are presented as mean ± SEM. *No.*, number; *TCN*, total cell number

### Effect of vitrification on the levels of demethylase and methyltransferases in embryos

**Fig. 4:**
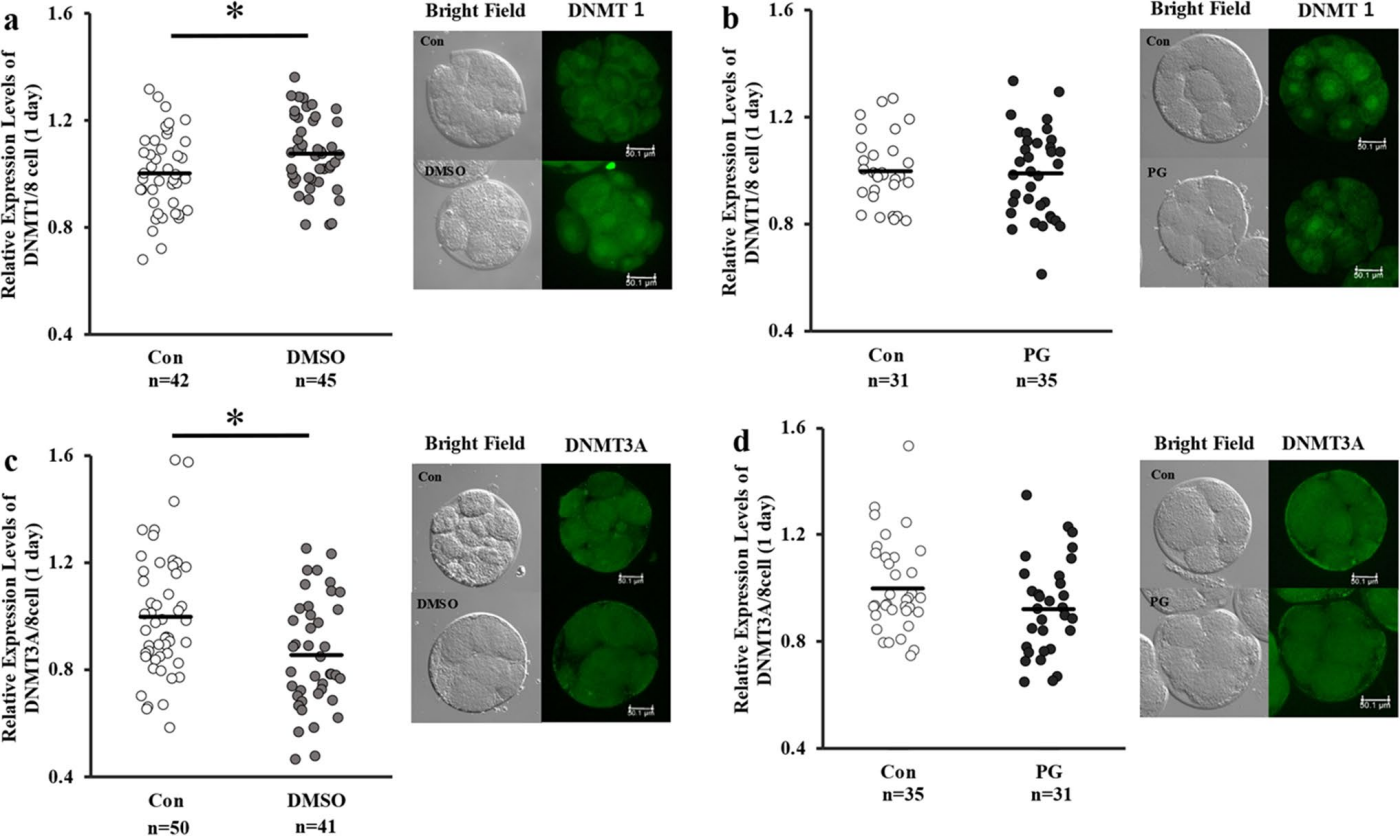
Expression levels of DNMTs in fresh (control) or vitrified-warmed embryos. Eight-cell stage embryos were vitrified using DMSO + EG or PG + EG and cultured for 1 day, and the relative expression levels of DNMT1 (**a**, **b**) and DNMT3A (**c**, **d**) were determined. Average of control was defined as 1.0. *n* indicates the number of embryos used. Representative image is shown beside each figure. The bar indicates 50 µm. *P < 0.05. a, b: *t* test was used for the comparison. c, d: Mann–Whitey *U* test was used for the comparison

**Fig. 5:**
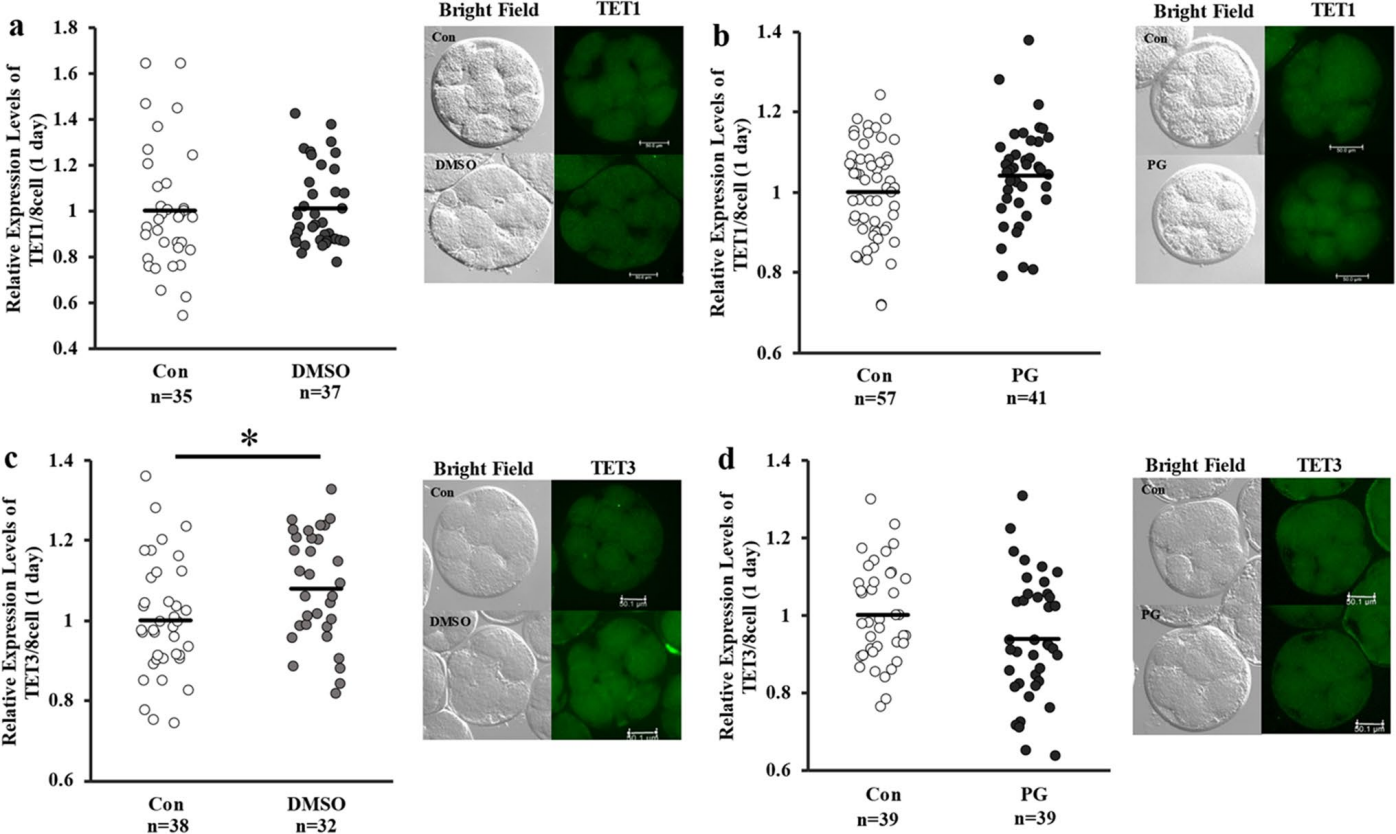
Expression levels of TETs in fresh (control) or vitrified-warmed embryos. Eight-cell stage embryos were vitrified using DMSO + EG or PG + EG and cultured for 1 day, and the relative expression levels of TET1 (**a**, **b**) and TET3 (**c**, **d**) were determined. Average of control was defined as 1.0. *n* indicates the number of embryos used. Representative image is shown beside each figure and the bar indicate 50 µm. *P < 0.05. a, b, d: *t* test was used for the comparison. c: Mann–Whitey *U* test was used for the comparison

**Fig. 6:**
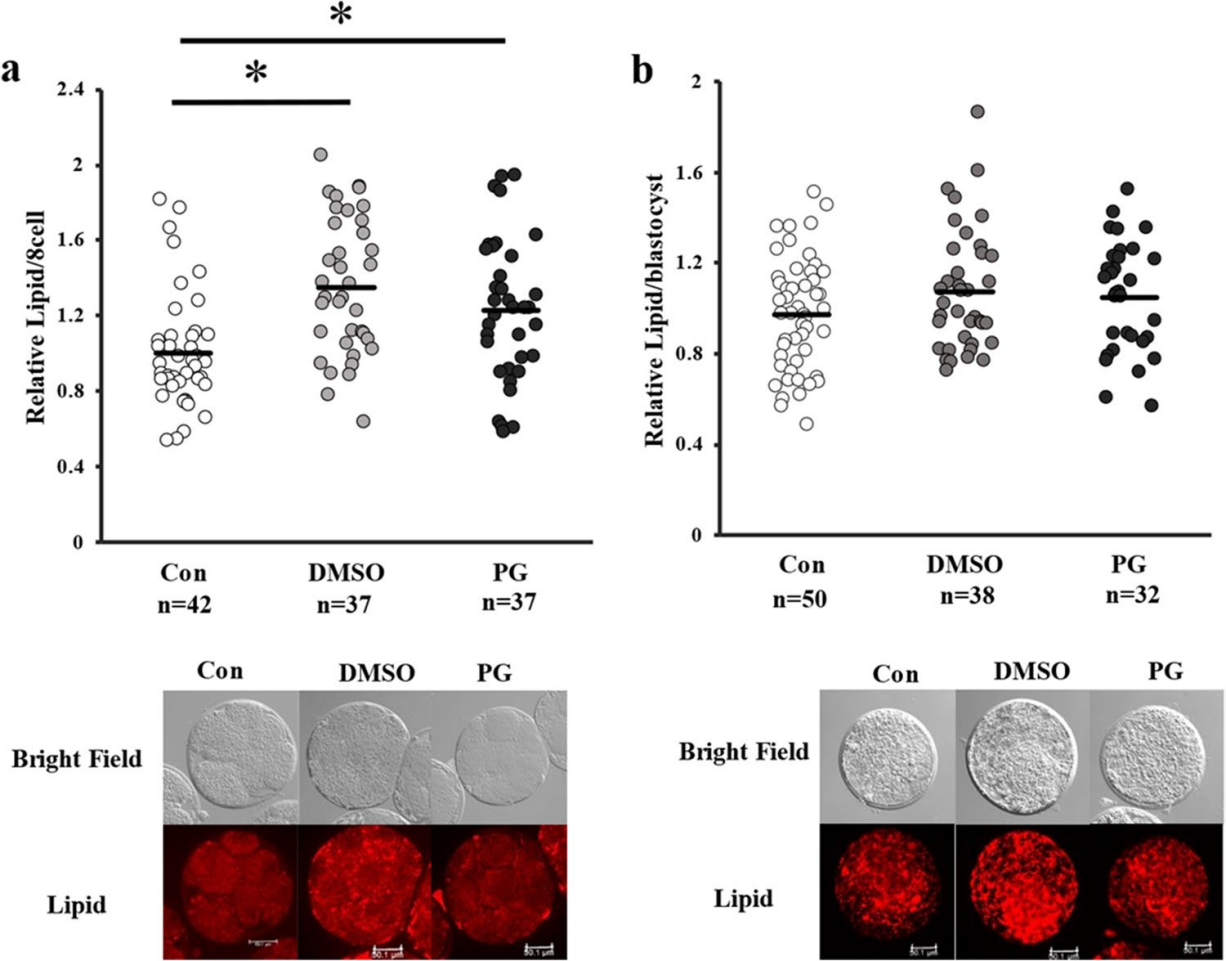
Effect of DMSO + EG or PG + EG vitrification on the lipid content of embryos. Vitrified-warmed embryos were cultured for 1 or 5 days when embryos (**a**) and blastocysts (**b**) were examined. Control indicates fresh embryos. Average of control was defined as 1.0. *n* indicates the number of embryos used. Representative image is shown below the figures and the bar indicates 50.1 µm. *P < 0.05. a: Kruskal–Wallis test was used for the comparisons. b: After one-way analysis of variance analysis, a post hoc test was used for the comparisons

The expression levels of TET1 in the fresh and vitrified-warmed embryos were comparable, whereas the expression levels of DNMT3A were significantly lower, and those of DNMT1 and TET3 were significantly higher in the DMSO + EG group than that in the control group (Figs. 4 and 5). As shown in Fig. 6a and b, the lipid content in the vitrified-warmed 8-cell stage embryos was significantly higher than that in the control groups, but non-significantly different from than at the blastocyst stage (Fig. 6).

### RNA-seq

RNA-seq analysis revealed that DEGs were induced upon treatment with a combination of PG + EG or DMSO + EG. Pathways enriched by the DEGs (679 in number) in the DMSO group includes the cytoskeleton in muscle cells, cell adhesion molecules, MAPK signaling pathway, cGMP-PKG signaling pathway, and PI3K-Akt signaling pathway (Table 4). The DEGs (1095 in number) in the PG group included ribosomes, oxidative phosphorylation, and thermogenesis (Table 5). The genes overlapping between the two groups (258 in number) were associated with insulin secretion, cytoskeleton in muscle cells, the cGMP-PKG signaling pathway, and the Wnt signaling pathway (Table 6). Furthermore, the DEGs between the fresh control and DMSO + EG groups were clustered into eight groups using the *K-medoids* methods (Fig. 1a) considering the direction of expression of the PG group against that of the DMSO group. Gene groups with strong adverse directional changes between the PG and DMSO groups (PG/DMSO) compared to the changes between the DMSO and control groups (DMSO/control) were extracted. These genes (144 in number) were associated with MAPK signaling and cell adhesion molecules (Fig. 1b). Furthermore, GO analysis of the genes showed that extracellular matrix, cell junction, and cell surface were significant cellular component (Table 7).

**Table 4:** KEGG pathway enriched by differentially expressed genes between DMSO and fresh groups

Term	Count	*P* value
Cytoskeleton in muscle cells	20	7.6E-05
Cell adhesion molecules	14	0.002
Transcriptional misregulation in cancer	15	0.003
MAPK signaling pathway	19	0.004
cGMP-PKG signaling pathway	13	0.006
PI3K-Akt signaling pathway	21	0.015
TGF-beta signaling pathway	9	0.017
Complement and coagulation cascades	8	0.022
Aldosterone synthesis and secretion	8	0.026
Malaria	6	0.028
Calcium signaling pathway	15	0.029
AGE-RAGE signaling pathway in diabetic complications	8	0.035
Fluid shear stress and atherosclerosis	10	0.038
Ovarian steroidogenesis	6	0.041

**Table 5:** KEGG pathway enriched by differentially expressed genes between PG and fresh groups

Term	Count	*P* value
Ribosome	49	1.3E-19
Oxidative phosphorylation	41	7.3E-17
Thermogenesis	49	8.2E-15
Coronavirus disease −19	52	2.9E-13
Prion disease	50	7.3E-13
Parkinson disease	50	8.2E-13
Diabetic cardiomyopathy	43	1.0E-12
Huntington disease	52	4.6E-12
Chemical carcinogenesis: reactive oxygen species	44	1.4E-11
Alzheimer’s disease	57	7.1E-11
Pathways of neurodegeneration: multiple diseases	60	5.5E-09
Amyotrophic lateral sclerosis	50	3.4E-08
Retrograde endocannabinoid signaling	26	3.4E-07
Non-alcoholic fatty liver disease	27	1.8E-06
Metabolic pathways	108	0.006
HIF-1 signaling pathway	13	0.013
Oocyte meiosis	14	0.016
Cardiac muscle contraction	12	0.022
Platelet activation	13	0.025
Phospholipase D signaling pathway	15	0.030
Pathways in cancer	40	0.034
Parathyroid hormone synthesis, secretion, and action	12	0.037

**Table 6:** Significantly enriched KEGG pathways obtained by overlapping differentially expressed genes between the DMSO and PG groups

Term	Count	*P* value
Adrenergic signaling in cardiomyocytes	7	0.005
Insulin secretion	5	0.011
Aldosterone synthesis and secretion	5	0.016
Parathyroid hormone synthesis, secretion and action	5	0.029
Cholinergic synapse	5	0.029
Cytoskeleton in muscle cells	7	0.030
cGMP-PKG signaling pathway	6	0.030
Wnt signaling pathway	6	0.035
Inositol phosphate metabolism	4	0.038

**Table 7:** DEGs in the cellular component group following GO analysis

Term	Count	*P* value
Extracellular space	23	6.3E-05
Extracellular matrix	7	0.0004
Cell junction	4	0.008
Intercalated disc	3	0.010
Cell surface	7	0.015
Early endosome	5	0.028
Fascia adherens	2	0.034
Nucleoplasm	16	0.041

### Effect of supplementation of vitrification medium containing DMSO with NAC on 5mC levels in 8-cell and blastocysts stage embryos

**Fig. 7:**
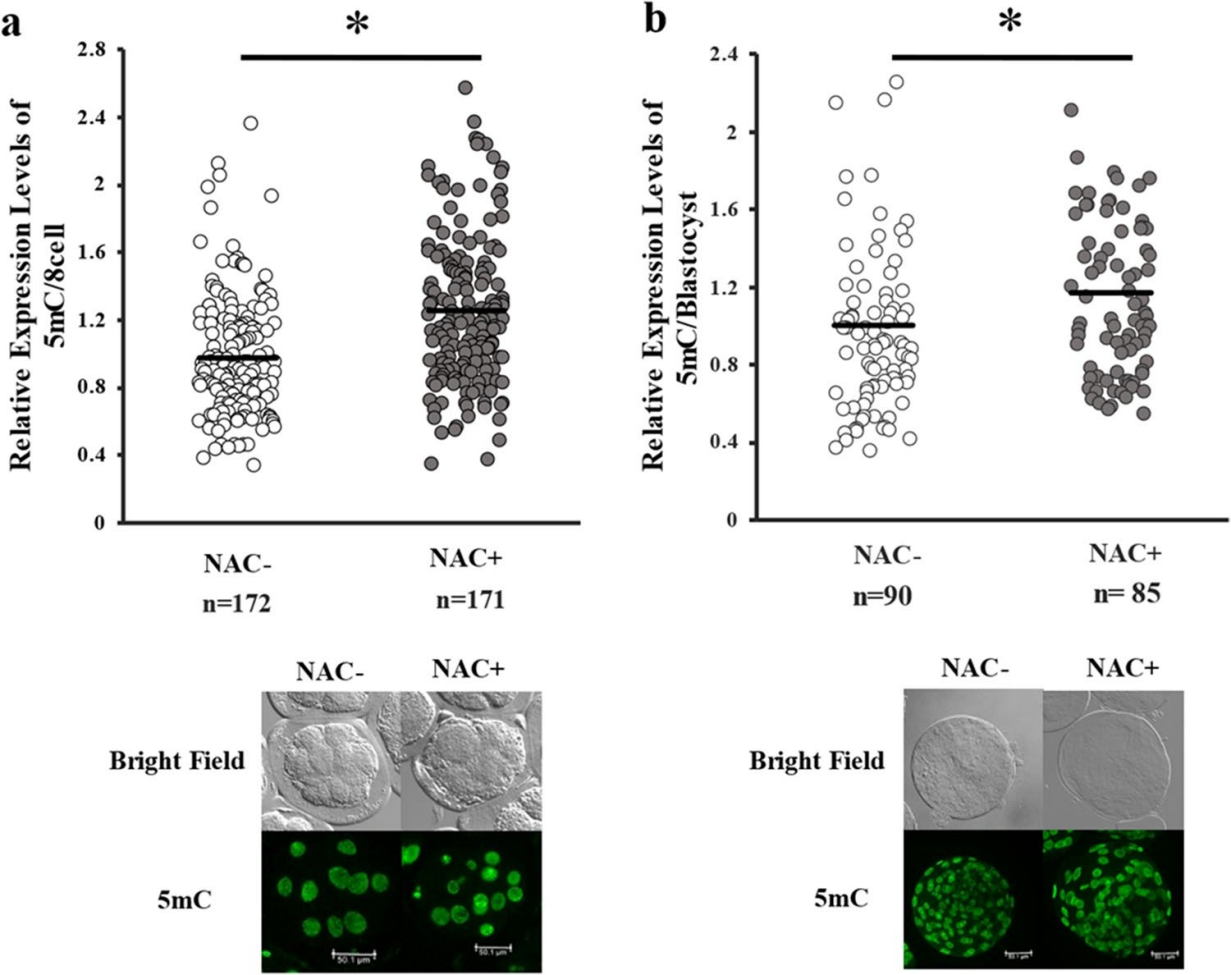
Effect of N-acetyl-l-cysteine supplementation on DMSO-induced DNA demethylation in vitrified-warmed embryos. 5mC expression levels in blastomere of embryos (**a**) and blastocysts (**b**). Control indicates fresh embryos. Average of control was defined as 1.0. *n* indicates the number of samples used. Representative image is shown below the figures. *P < 0.05. Number of embryos: 20 and 20 for a; 17 and 18 for b, respectively. a, b: Mann–Whitey *U* test was used for comparison

The addition of NAC did not affect the developmental rate or total cell number of the blastocysts (Table 8). After vitrification in the presence of NAC, the levels of DNA methylation in both the 8-cell stage and blastocysts were lower than in those without NAC treatment (Fig. 7).

**Table 8:** Effect of addition of vitrification solution with NAC on embryonic development

NAC (5 mM)	No. of	No. of	Blastocysts		
	Trials	embryos	Rate (%)	No	TCN
** − **	14	140	26.4 ± 3.6	21	111 ± 10.0
+	13	130	25.3 ± 5.3	15	94 ± 8.0

Eight cell stage embryos were vitrified using DMSO + EG (NAC** −**) and DMSO + EG + NAC 5 mM (NAC +). Date are presented as mean ± SEM. *No.*, number; *TCN* total cell number

## Discussion

This study demonstrated that vitrification with DMSO lowered DNA methylation levels at the blastocyst stage. Vitrification with PG increased DNA methylation, and the results of immunostaining and RNA-seq analyses revealed differential effects of the two cryoprotectants. In addition, the addition of NAC to the vitrification medium containing DMSO ameliorated vitrification-induced demethylation in warmed embryos.

Reports show that vitrification of mouse and bovine oocytes alters DNA methylation levels in embryos [11, 17, 18]. In addition, the vitrification of early cleaved-stage embryos with DMSO results in low levels of DNA methylation [10]. Consistent with these reports, the present study showed that the DNA methylation levels of blastocysts derived from 8-cell stage embryos vitrified with DMSO + EG were lower than those from fresh embryos. In contrast, vitrification of super-ovulated oocytes with DMSO results in low DNA methylation in the ICM of blastocysts [19]. However, the contribution of background differential methylation of the ICM and TE in vitrified-warmed embryos remain unclear.

The present study showed that the use of PG instead of DMSO during vitrification did not increase DNA methylation in the blastocysts. Weihong et al. reported that vitrification of bovine oocytes with PG induced high levels of DNA methylation in blastocysts [11]. In addition, Pollock et al. demonstrated that cryopreservation-induced DNA methylation was ameliorated when osmolytes such as sucrose and glycerol were used instead of DMSO for the cryopreservation of mesenchymal stromatolites cells [12]. Based on these reports, we concluded that DMSO is a major causal factor for DNA demethylation in embryos. Embryonic DNA methylation is regulated by demethylases (TETs) and methyltransferases (DNMTs). Several studies have reported altered levels of DNMTs and TETs in vitrified-warmed embryos; the expression levels of DNMT3A in blastocysts derived from vitrified mouse oocytes were lower than those of their fresh counterparts [20], and Ma et al. [21] reported low levels of DNMT1 in vitrified-warmed mouse oocytes. In contrast, high levels of TET1 were detected in 4–8-cell stage embryos derived from vitrified-warmed oocytes [22], and high levels of TET3 were present in vitrified-warmed mouse oocytes [23]. In the present study, high levels of DNMT1 and TET3 and low levels of DNMT3A were detected in the 8-cell stage embryos vitrified using DMSO. Considering that vitrification increased the level of 5hmC in embryos, we suggested that TET3 may play a role in the reduction of 5mC levels in the DMSO group embryos; however, the mechanism underlying the variation in the expression levels of DNMTs and TETs in vitrified-warmed embryos remains unclear. Fu et al. [23] reported that vitrification resulted in high TET3 levels in warmed oocytes; however, modification of TET3 activity using vitamin C and an antagonist of alfa-ketoglutarate did not affect 5mC levels, suggesting that other factors may contribute to the low 5mC levels in vitrified-warmed embryos.

DMSO, a well-known potent oxidant and source of oxygen [24], supports C-H hydroxylation [25]. In addition, DMSO converts cystatin to cystine [26]. In the present study, the addition of NAC to the vitrification medium alleviated the extent of reduction in 5mC levels in the 8-cell and blastocyst stage embryos derived from vitrified-warmed embryos, indicating that the SH residues in NAC hinder the action of DMSO on methyl residues in DNA; however, precise validation is needed to verify this hypothesis. Furthermore, NAC is a possible antagonist of DMSO-induced demethylation. Consistently, NAC attenuated oxidative stress in human nucleus pulposus cells cryopreserved in DMSO [27].

Studies have shown that embryo vitrification affects several groups of genes in sheep [28] and induces major transcriptomic changes in human oocytes [29]. We conducted RNA-seq analysis to determine the differential effects of DMSO and PG vitrification on warmed embryos. The ribosome and oxidative phosphorylation pathway were affected in only the PG-treated embryos, which was the major difference between the PG- and DMSO-induced gene expression profiles. These results suggests that PG affects mitochondria and protein synthesis in embryos, while accumulating evidence has shown that vitrification affects mitochondrial integrity and functions [30]. The present analysis showed that pathways enriched by DEGs between the DMSO + EG and fresh embryo groups included the MAPK and PI3K-Akt signaling pathways. These pathways have been reported to be affected by EG [31]. DEGs overlapping between the DMSO-induced DEGs and PG-induced DEGs were associated with insulin secretion, cytoskeleton in muscle cells, and cGMP-PKG and Wnt signaling. Consistent with our observations, Wiltshire et al. [32] have reported that the Wnt and insulin secretory pathway are affected by the vitrification of human oocytes. These results indicate that vitrification itself affects this pathway in warmed embryos. cGMP-PKG signaling is associated with lipid metabolism and increased cGMP activity in oocytes, resulting in lower lipid content in oocytes [33]. In agreement with this report, we showed that vitrification significantly increased lipid content in vitrified embryos irrespective of the cryoprotectants used, implying that vitrification may alter cGMP levels, affecting the lipid content in embryos. To obtain insights regarding the differences between DMSO and PG, we used the *K-medoids* method where DEGs having differential directional change in PG group against DMSO group are selected from the clustered DEGs. Gene groups with adverse directional change between DMSO and PG were enriched for cell adhesion and MAPK signaling. Furthermore, GO analysis of the gene groups showed that these gene groups were enriched on the cell surface and cellular matrix, similar to that in the cellular compartment category of GO analysis. These results indicate that DMSO affects the cell surface, cell adhesion, and MAPK signaling.

DMSO has been widely used as a vehicle and cryoprotectant because of its low toxicity. However, even low concentrations of DMSO have been shown to alter gene expression in cardiac tissues [34] and induce cellular differentiation by altering DNA methylation. Therefore, alternative cryoprotectants or molecules that alleviate DMSO-induced demethylation should be investigated. We propose that NAC and PG may possess protective properties. Accumulating evidence show that neonatal birth weight was higher for frozen embryo transfer than that for fresh embryo transfer [35, 36]. In addition, the weight of the children from frozen embryo transfer was heavier than fresh counterparts up to 6 years old [37]. In contrast, the difference during infancy and childhood is not significant [36]. In the report, authors suggest that the differential birth weight for frozen embryo transfer is due to cycle protocol used. In this study, we show that differentially expressed genes and differential DNA methylation in vitrified-warmed embryos, which imply that vitrification induced epigenetic changes may affect birth weight of calves. We did not examine the long-term consequence of vitrification, which is a limitation of this study. Therefore, further studies in this regard are required.

In conclusion, vitrification with DMSO reduced 5mC levels in blastocysts; in contrast, PG did not decrease 5mC levels but affected DNA methylation. The addition of NAC to the vitrification medium alleviated the effect of DMSO on 5mC in vitrified-warmed embryos, suggesting NAC is a potential antagonist of DMSO-induced demethylation. RNA-seq analysis revealed that PG had a greater effect on genes related to the mitochondria and ribosome, while DMSO affected the cellular surface, adhesion, and MAPK signaling pathways.

## Data Availability

All data will be provided by the corresponding author at a proper request.

## References

[1] Dalcin L, Silva RC, Paulini F, Silva BD, Neves JP, Lucci CM. Cytoskeleton structure, pattern of mitochondrial activity and ultrastructure of frozen or vitrified sheep embryos. Cryobiology. 10.1016/j.cryobiol.2013;67(2):137-145. 10.1016/j.cryobiol.2013.05.012 23770514

[2] Amoushahi M, Salehnia M, Mowla SJ. Vitrification of mouse MII oocyte decreases the mitochondrial DNA copy number, TFAM gene expression and mitochondrial enzyme activity. J Reprod Infertil. 2017;18(4):343–51. 29201664 PMC5691250

[3] Lei T, Guo N, Liu JQ, Tan MH, Li YF. Vitrification of in vitro matured oocytes: effects on meiotic spindle configuration and mitochondrial function. Int J Clin Exp Pathol. 2014;7(3):1159–65. 24696732 PMC3971321

[4] Xu J, Sun L, Wu C, Zhang S, Ju S, Rui R, Zhang D, Dai J. Theriogenology Involvement of PINK1/Parkin-mediated mitophagy in mitochondrial functional disruption under oxidative stress in vitrified porcine oocytes. Theriogenology. 2021;174:160–8. 10.1016/j.theriogenology.2021.08.028. 34455243 10.1016/j.theriogenology.2021.08.028

[5] Somfai T, Haraguchi S, Dang-Nguyen TQ, Kaneko H, Kikuchi K. Vitrification of porcine immature oocytes and zygotes results in different levels of DNA damage which reflects developmental competence to the blastocyst stage. PLoS ONE. 2023;18(3):e0282959. 10.1371/journal.pone.0282959. 36930621 10.1371/journal.pone.0282959 PMC10022796

[6] Zhang J, Wang Y, Liu H, Mao X, Chen Q, Fan Y, et al. Effect of in vitro culture period on birth weight after vitrified-warmed transfer cycles: analysis of 4,201 singleton newborns. Fertil Steril. 2019;111(1):97–104. 10.1016/j.fertnstert.2018.10.006. 30458993 10.1016/j.fertnstert.2018.10.006

[7] Beyer DA, Griesinger G. Vitrified-warmed embryo transfer is associated with mean higher singleton birth weight compared to fresh embryo transfer. Eur J Obstet Gynecol Reprod Biol. 2016;203:104–7. 10.1016/j.ejogrb.2016.05.041. 27267871 10.1016/j.ejogrb.2016.05.041

[8] Maris E, Ferrieres-Hoa A, Gala A, Coffy A, Vintejoux E, Ranisavljevic N, et al. Embryons vitrifiés, embryons frais : comparaison des poids de naissance [Comparison of birth weights of children born after slow frozen embryo replacement versus fresh embryo transfer]. Gynecol Obstet Fertil Senol. 2019;47(3):305–10. 10.1016/j.gofs.2019.01.011. 30745159 10.1016/j.gofs.2019.01.011

[9] Kwan HCK. Reconsideration of the safety and effectiveness of human oocyte cryopreservation. Reprod Biol Endocrinol. 2023;21(1):22. 10.1186/s12958-023-01071-z. 36849982 10.1186/s12958-023-01071-z PMC9969709

[10] Yao J, Geng L, Huang R, Peng W, Chen X, Jiang X, et al. Effect of vitrification on in vitro development and imprinted gene Grb10 in mouse embryos. Reproduction. 2017;154(3):97–105. 10.1530/REP-16-0480. 28696244 10.1530/REP-16-0480

[11] Hu W, Marchesi D, Qiao J, Feng HL. Effect of slow freeze versus vitrification on the oocyte: an animal model. Fertil Steril. 2012;98(3):752-760.e3. 10.1016/j.fertnstert.2012.05.037. 22766176 10.1016/j.fertnstert.2012.05.037

[12] Pollock K, Samsonraj RM, Dudakovic A, Thaler R, Stumbras A, McKenna DH, et al. Improved post-thaw function and epigenetic changes in mesenchymal stromal cells cryopreserved using multicomponent osmolyte solutions. Stem Cells Dev. 2017;26(11):828–42. 10.1089/scd.2016.0347. 28178884 10.1089/scd.2016.0347 PMC5466057

[13] Cheng H, Han Y, Zhang J, Zhang S, Zhai Y, An X, et al. Effects of dimethyl sulfoxide (DMSO) on DNA methylation and histone modification in parthenogenetically activated porcine embryos. Reprod Fertil Dev. 2022;34(8):598–607. 10.1071/RD21083. 35397781 10.1071/RD21083

[14] Hara S, Aoki S, Nagata M, Shirasuna K, Noguchi T, Iwata H. Xanthan gum and locust bean gum substrate improves bovine embryo development. Reprod Domest Anim. 2020;55(9):1124–31. 10.1111/rda.13750. 32562321 10.1111/rda.13750

[15] Aoki S, Inoue Y, Shinozawa A, Tanaka K, Shirasuna K, Iwata H. miR-17-5p in bovine oviductal fluid affects embryo development. Mol Cell Endocrinol. 2022;551:111651. 10.1016/j.mce.2020.111651. 35452772 10.1016/j.mce.2022.111651

[16] Kaufman L, Rousseeuw PJ. Finding groups in data: an introduction to cluster analysis. Wiley Serries in Probability and Statistics. 1990. John Wiley & Sons, Inc.

[17] Liang Y, Fu XW, Li JJ, Yuan DS, Zhu SE. DNA methylation pattern in mouse oocytes and their in vitro fertilized early embryos: effect of oocyte vitrification. Zygote. 2014;22(2):138–45. 10.1017/S0967199412000512. 23174120 10.1017/S0967199412000512

[18] Yodrug T, Parnpai R, Hirao Y, Somfai T. Effect of vitrification at different meiotic stages on epigenetic characteristics of bovine oocytes and subsequently developing embryos. Anim Sci J. 2021;92(1):e13596. 10.1111/asj.13596. 34309122 10.1111/asj.13596

[19] Bakhtari A, Rahmani HR, Bonakdar E, Jafarpour F, Asgari V, Hosseini SM, et al. The interfering effects of superovulation and vitrification upon some important epigenetic biomarkers in mouse blastocyst. Cryobiology. 2014;69(3):419–27. 10.1016/j.cryobiol.2014.09.379. 25307438 10.1016/j.cryobiol.2014.09.379

[20] Cheng KR, Fu XW, Zhang RN, Jia GX, Hou YP, Zhu SE. Effect of oocyte vitrification on deoxyribonucleic acid methylation of H19, Peg3, and Snrpn differentially methylated regions in mouse blastocysts. Fertil Steril. 2014;102(4):11831190.e3. 10.1016/j.fertnstert.2014.06.037. 10.1016/j.fertnstert.2014.06.037 25064401

[21] Ma Y, Long C, Liu G, Bai H, Ma L, Bai T, et al. WGBS combined with RNA-seq analysis revealed that Dnmt1 affects the methylation modification and gene expression changes during mouse oocyte vitrification. Theriogenology. 2022;177:11–21. 10.1016/j.theriogenology.2021.09.032. 34653792 10.1016/j.theriogenology.2021.09.032

[22] Gao J, Wang Y, Guan YM, Chen CQ. Fusarium cerealis, a new pathogen causing ginseng (Panax ginseng) root rot in China. Plant Dis. 2014;98(10):1433. 10.1094/PDIS-03-14-0328-PDN. 30703961 10.1094/PDIS-03-14-0328-PDN

[23] Fu L, Chang H, Wang Z, Xie X, Chen H, Lei Z, et al. The effects of TETs on DNA methylation and hydroxymethylation of mouse oocytes after vitrification and warming. Cryobiology. 2019;90:41–6. 10.1016/j.cryobiol.2019.09.001. 31513810 10.1016/j.cryobiol.2019.09.001

[24] Liang YF, Wu K, Song S, Li X, Huang X, Jiao N. I2- or NBS-catalyzed highly efficient α-hydroxylation of ketones with dimethyl sulfoxide. Org Lett. 2015;17(4):876–9. 10.1021/ol5037387. 25650782 10.1021/ol5037387

[25] Borchgrevink PC. Behandling av kreftrelatert smerte [Treatment of cancer-related pain]. Tidsskr Nor Laegeforen. 1989;109(33):3399–400. 2481892

[26] Zhang Q, Chen C, Weng C, Chen J, Peng Z, Lin Q, et al. Oxidation analysis of l-cysteine with a chiral sensor based on quantum weak measurement. Anal Chem. 2024;96(8):3402–8. 10.1021/acs.analchem.3c04558. 38355418 10.1021/acs.analchem.3c04558

[27] Tamagawa S, Sakai D, Schol J, Sako K, Nakamura Y, Matsushita E, et al. *N*-acetylcysteine attenuates oxidative stress-mediated cell viability loss induced by dimethyl sulfoxide in cryopreservation of human nucleus pulposus cells: a potential solution for mass production. JOR Spine. 2022;5(4):e1223. 10.1002/jsp2.1223. 36601378 10.1002/jsp2.1223 PMC9799083

[28] Brair VL, Maia ALRS, Correia LFL, Barbosa NO, Santos JDK, Brandão FZ, et al. Gene expression patterns of in vivo-derived sheep blastocysts is more affected by vitrification than slow freezing technique. Cryobiology. 2020;95:110–5. 10.1016/j.cryobiol.2020.05.009. 32554154 10.1016/j.cryobiol.2020.05.009

[29] Semenova EA, Hall ECR, Ahmetov II. Genes and athletic performance: the 2023 update. Genes (Basel). 2023;14(6):1235. 10.3390/genes14061235. 37372415 10.3390/genes14061235 PMC10298527

[30] Lei T, Guo N, Tan MH, Li YF. Effect of mouse oocyte vitrification on mitochondrial membrane potential and distribution. J Huazhong Univ Sci Technolog Med Sci. 2014;34(1):99–102. 10.1007/s11596-014-1238-8. 24496686 10.1007/s11596-014-1238-8

[31] Ji P, Liu Y, Yan L, Jia Y, Zhan M, Lv D, et al. Melatonin improves the vitrification of sheep morulae by modulating transcriptome. Front Vet Sci. 2023;10:1212047. 10.3390/genes14061232. 37920328 10.3389/fvets.2023.1212047 PMC10619913

[32] Wiltshire A, Schaal R, Wang F, Tsou T, McKerrow W, Keefe D. Vitrification with dimethyl sulfoxide induces transcriptomic alteration of gene and transposable element expression in immature human oocytes. Genes (Basel). 2023;14(6):1232. 10.3390/genes14061232. 37372413 10.3390/genes14061232 PMC10298107

[33] Schwarz KRL, de Castro FC, Schefer L, Botigelli RC, Paschoal DM, Fernandes H et al. The role of cGMP as a mediator of lipolysis in bovine oocytes and its effects on embryo development and cryopreservation [published correction appears in PLoS One. 2018;13(4):e0196268. 10.1371/journal.pone.0191023 10.1371/journal.pone.0191023 PMC5779671 29360833

[34] Verheijen M, Lienhard M, Schrooders Y, Clayton O, Nudischer R, Boerno S, et al. DMSO induces drastic changes in human cellular processes and epigenetic landscape in vitro. Sci Rep. 2019;9(1):4641. 10.1038/s41598-019-40660-0. 30874586 10.1038/s41598-019-40660-0 PMC6420634

[35] Zhang J, Du M, Li Z, Wang L, Hu J, Zhao B, et al. Fresh versus frozen embryo transfer for full-term singleton birth: a retrospective cohort study. J Ovarian Res. 2018;11(1):59. 10.1186/s13048-018-0432-x. 30012201 10.1186/s13048-018-0432-x PMC6048709

[36] Belva F, Blockeel C, Keymolen K, Buysse A, Bonduelle M, Verheyen G, et al. Impact of embryo vitrification on children’s health, including growth up to two years of age, in comparison with results following a fresh embryo transfer. Fertil Steril. 2023;119(6):932–41. 10.1016/j.fertnstert.2023.02.006. 36774979 10.1016/j.fertnstert.2023.02.006

[37] Magnus MC, Wilcox AJ, Fadum EA, Gjessing HK, Opdahl S, Juliusson PB, et al. Growth in children conceived by ART. Hum Reprod. 2021;36(4):1074–82. 10.1093/humrep/deab007. 33592626 10.1093/humrep/deab007 PMC7970724

